# C/EBPβ-Thr217 Phosphorylation Stimulates Macrophage Inflammasome Activation and Liver Injury

**DOI:** 10.1038/srep24268

**Published:** 2016-04-12

**Authors:** Martina Buck, Jose Solis-Herruzo, Mario Chojkier

**Affiliations:** 1 Department of Medicine, University of California, San Diego, La Jolla, CA, USA; 2 Veterans Affairs San Diego Healthcare System, San Diego, CA, USA; 3 Biomedical Sciences Program, University of California, San Diego, La Jolla, CA, USA; 4 Department of Medicine, Universidad Complutense de Madrid, Madrid, Spain; 5 Clinical Translational Research Institute, University of California, San Diego, La Jolla, CA, USA

## Abstract

Amplification of liver injury is mediated by macrophages but the signaling by which the macrophage inflammasome enhances liver injury is not completely understood. The CCAAT/Enhancer Binding Protein-β (C/EBPβ) is a critical signaling molecule for macrophages because expression of a dominant inhibitor of C/EBPβ DNA-binding sites or a targeted deletion of C/EBPβ results in impaired macrophage differentiation. We reported that expression of the phosphorylation-mutant C/EBPβ-Glu217, which mimics phosphorylated C/EBPβ-Thr217, was sufficient to confer macrophage survival to Anthrax lethal toxin. Here, using primary hepatocytes, primary liver macrophages, dominant positive and negative transgenic mice of the C/EBPβ-Thr217 phosphoacceptor, macrophage ablation, and an inhibitory peptide of C/EBPβ-Thr217 phosphorylation, we determined that this phosphorylation is essential for the activation of the inflammasome in liver macrophages and for the hepatocyte apoptosis induced by hepatotoxins that results in liver injury. Similar findings were observed in the livers of patients with acute injury induced by Toxic Oil Syndrome.

Inflammation contributes to the pathogenesis of most acute and chronic liver diseases^1^. Excessive liver injury and inflammation associated with liver diseases induced by viral, toxic, immunologic, and metabolic diseases^2^ results in liver dysfunction and in chronic conditions in the potential deposition of scar tissue and the development of cirrhosis, which is in turn a major contributor to the morbidity and mortality of patients affected by chronic liver diseases^2^,^3^. We first reported that amplification of toxic liver injury is mediated by macrophages since TLR-4 ko mice were resistant to hepatotoxins and that reconstitution of bone marrow irradiated TLR-4 ko mice with TLR-4^+/+^ macrophages conferred susceptibility of these animals to hepatotoxins^4^. The role of macrophages in liver inflammation in toxic liver injury has been confirmed using macrophage ablation^5^, and further characterized in an experimental alcoholic liver injury model using an IL-1 receptor antagonist^6^, and in LPS/D-galactosamine induced liver injury using Adenosine-_2A_ (A_2A_) receptor-ko mice^7^. Fas–mediated IL-18 secretion from macrophages causes acute liver injury in mice^8^, and macrophage phagocytosis removes hepatocyte debris during hepatocyte injury^9^. However, the signal transduction mechanisms in liver macrophages that are indispensable to amplify liver injury have been only partially characterized^1^.

The inflammasome is a protein complex that is essential for triggering activation of inflammatory reactions in macrophages as well as the consequent macrophage activation^1^,^10^,^11^. The CCAAT/Enhancer Binding Protein-β (C/EBPβ)^12^,^13^,^14^ has been shown to be a critical signaling molecule for macrophages as expression of a dominant inhibitor of C/EBPβ DNA-binding sites^15^ or a targeted deletion of C/EBPβ results in impaired macrophage differentiation^16^.

In addition, C/EBPβ expression is dramatically increased during differentiation of these cells, and is induced by macrophage modulators (LPS, IL-1, G-CSF, TGFβ, vitamin D, retinoic acid)^13^,^17^. In this context, we and others have shown that phosphorylation of C/EBPβ by Ribosomal S-Kinase-2 (RSK-2), which is activated directly by Extracellular-Regulated Kinase (ERK)-1/2 phosphorylation, plays an essential role in the ERK/ Mitogen Activated Protein Kinase (MAPK) signaling pathway regulating cell survival^18^,^19^,^20^,^21^. Relevant to macrophage activation and survival, we have reported that expression of the dominant positive, phosphorylation-mutant C/EBPβ-Glu217, which mimics phosphorylated C/EBPβ-Thr217 in biological assays^22^, was sufficient to rescue the impaired macrophage function and activity induced by Anthrax lethal toxin^23^.

Knowledge of the specific signaling that targets a single amino acid within a specific phosphoacceptor domain of the mechanistic protein (in this case C/EBPβ) is necessary to understand the process of the disease and to eventually design effective targeted therapeutics that are still lacking in the treatment of human liver injury. Therefore, we investigated whether signaling through phosphorylation of C/EBPβ-Thr217, a potential novel therapeutic target, might be a major mechanism responsible for liver inflammation and injury through the activation of the inflammasome in liver macrophages. We studied the effects of C/EBPβ-Phospho-Thr217 signaling that is evolutionarily conserved (identical in human C/EBPβ-Phospho-Thr266) on macrophage inflammasome activity and liver injury induced by hepatotoxins in mice and humans.

## Results

### The modulation of Fas-L induced liver injury and inflammation by phosphorylated C/EBPβ-Thr217 in mice

We determined the degree of liver injury after exposure to hepatotoxins (Fas and CCl_4_) in mice by quantitative histology and immunohistochemistry^24^, cell death assays^23^, and by measuring serum alanine aminotransferase (ALT) levels^21^, an indicator of liver injury used routinely in patient care as well as by the Food and Drug Administration in clinical drug studies^25^.

**Figure 1:**
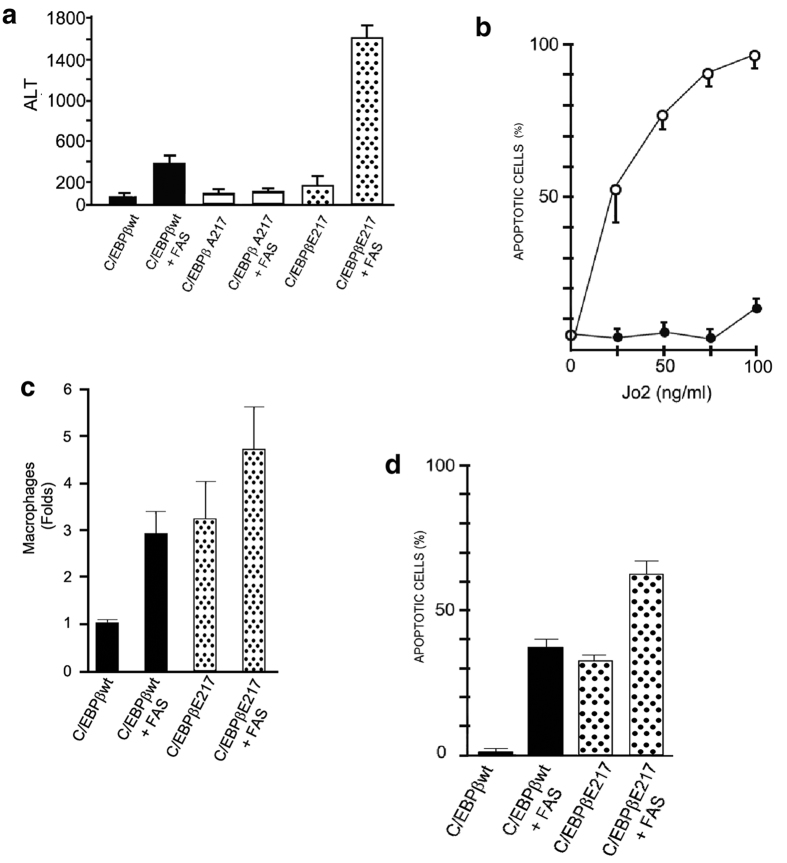
The modulation of Fas-L induced liver injury and inflammation by phosphorylated C/EBPβ-Thr217 in mice. (**a)** Serum ALT (IU/ml) levels were determined 12 hours after a single IP dose of Jo-2 Ab (FasL). Mice expressing the phosphorylation mimic C/EBPβ-Glu217 transgene were more susceptible than control C/EBPβ-wt mice to liver injury induced with Jo-2 Ab, judging by the serum ALT levels (*P* < 0.0001). Mice expressing the non-phosphorylatable, C/EBPβ-Ala217 transgene were highly resistant to Fas-L induction of liver injury (*P* < 0.01); n = 20 mice per group. (**b)** Jo-2 Ab induced minimal injury to cultured primary hepatocytes isolated from the phosphorylation mimic C/EBPβ-Glu217 transgenic mice (closed circles) when compared to hepatocytes from C/EBPβ-wt mice (open circles), judging by the apoptosis annexin-V assay (*P* < 0.001). Control cultured primary hepatocytes from C/EBPβ-wt untreated with Jo-2 had less than 5% baseline apoptosis. (**c)** Jo-2 Ab stimulated a greater infiltration of F4/80+ macrophage inflammatory cells in the livers of C/EBPβ-Glu217 mice than in the livers of C/EBPβ-wt mice (*P* < 0.01). (**d)** Jo-2 Ab induced a greater area of hepatocyte apoptotic damage in the livers of C/EBPβ-Glu217 mice than in the livers of C/EBPβ-wt mice (*P* < 0.005). Values are mean (SD) of at least 6 animals per group and representative of three experiments.

Fas-mediated IL-18 secretion by macrophages^8^ and injection of a Fas agonist antibody (Jo-2 Ab)^26^ induces severe liver injury in mice. First, we showed that mice expressing the dominant positive, phosphorylation mimic C/EBPβ-Glu217 transgene were more susceptible than control C/EBPβ-wt mice to liver injury induced by Fas-R activation with Jo-2 Ab, judging by the serum ALT levels (*P* < 0.0001) (Fig. 1a) and histology (Supplementary Fig. 1). Mice expressing the dominant negative, non-phosphorylatable, C/EBPβ-Ala217 transgene were highly resistant to Fas-L induction of liver injury (*P* < 0.01) (Fig. 1a). In contrast, Fas-L (Jo-2 Ab) induced minimal injury to cultured primary hepatocytes isolated from the phosphorylation mimic C/EBPβ-Glu217 transgenic mice when compared to hepatocytes from C/EBPβ-wt mice, judging by the apoptosis annexin-V assay (*P* < 0.001) (Fig. 1b). Control cultured primary hepatocytes from C/EBPβ-wt untreated with Jo-2 had less than 5% baseline apoptosis. Congruent with their resistance to Fas-induced cell injury, the C/EBPβ-Glu217 cultured primary hepatocytes were also refractory to apoptosis induced by the proteasome inhibitor lactacystin^27^ when compared to C/EBPβ-wt cultured primary hepatocytes (Supplementary Fig. 2). Collectively, these experiments indicate that the susceptibility to severe liver injury induced by Fas-L signaling requires phosphorylation of C/EBPβ-Thr217 in liver cells other than hepatocytes that would be missing from these tissue culture studies. Although of interest, the resistance of C/EBPβ-Glu217 hepatocytes to Fas and lactacystin induced injury is not the focus of these studies.

Both hepatocytes and non-parenchymal liver cells, including macrophages, express the Fas receptor (CD95)^28^ In this context, we found that Fas-L also stimulated a greater infiltration of F4/80+ macrophage inflammatory cells in the livers of C/EBPβ-Glu217 mice than in the livers of C/EBPβ-wt mice (Fig. 1c and Supplementary Fig. 1), which corresponded to a greater area of hepatocyte apoptotic damage (Fig. 1d and Supplementary Fig. 1).

### Activation of cultured primary liver macrophages by TGF-α is associated with phosphorylation of C/EBPβ-Thr217

The above experiments suggested that liver macrophages may contribute to the amplification of liver injury induced by Fas-L in C/EBPβ-Glu217 mice and be the general mechanism of injury in C/EBPβ-wt mice, as we and others reported for Fas-L and other animal models of liver injury^4^,^5^,^6^,^7^,^8^. Because expression of C/EBPβ in macrophages is of great relevance to the maturation and function of these cells^13^,^14^,^15^,^16^,^17^, we assessed whether phosphorylated C/EBPβ-Thr217 modulates the polarization of inflammatory primary liver macrophages, isolated as reported previously^23^.

**Figure 2:**
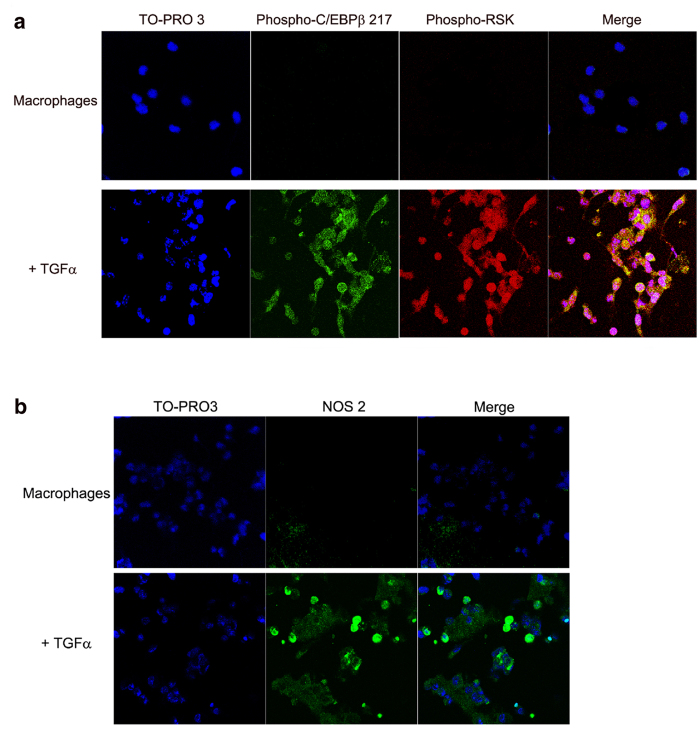
Activation of cultured primary liver macrophages by TGF-α is associated with phosphorylation of C/EBPβ-Thr217. Liver macrophages cultured in RPMI 1640, 10% fetal bovine serum with L-glutamine, 25 μM HEPES and Penicillin/Streptomycin were treated for 8 hr with TGFα (10 μM). (**a)** After treatment with TGF-α, freshly isolated cultured liver macrophages from C/EBPβ-wt mice expressed activated RSK-phospho-Ser380 and phosphorylation of endogenous C/EBPβ on Thr217 (*P* < 0.001). Representative examples of triplicate samples from three experiments. (**b)** TGF-α induced also expression of NOS-2 in cultured liver macrophages (*P* < 0.01). TO-PRO3 was used to stain cellular DNA. Representative examples of triplicate samples from three experiments. Fluorescence and bright-field imaging were quantified using the Keyence microscope BZ9000 analysis software programs.

After treatment with TGF-α, an activator of the MAPK signaling^18^ and a classical inflammatory macrophage inducer^17^, freshly isolated cultured liver macrophages from C/EBPβ-wt mice expressed activated RSK-phospho-Ser380 and phosphorylation of endogenous C/EBPβ on Thr217^18^ (Fig. 2a), as well as NOS-2, whose expression in activated macrophages is mediated by C/EBPβ^29^ (Fig. 2b). Collectively, these results indicate a potential link between phosphorylation of C/EBPβ-Thr217 in liver macrophages, macrophage activation and liver injury *in vivo* in mice and in cultured cells.

### Phosphorylation of C/EBPβ on Thr217 is induced and necessary for the liver macrophage activation after hepatotoxin treatment in mice

To analyze whether phosphorylation of C/EBPβ on Thr217 is induced and necessary for the liver macrophage activation by chemical liver injury, we administered a single dose of CCl_4_, which is a classical and predictable hepatotoxin that induces oxidative stress in rodent and human livers^21^,^30^,^31^, to C/EBPβ-wt, TGF-α, C/EBPβ-Glu217 and C/EBPβ-Ala217 transgenic mice. Eight hours later, C/EBPβ-wt mice received either an intraperitoneal injection of the cell permeant, dominant negative C/EBPβ peptide (100 μg) or vehicle (50 μl saline). In earlier studies, we found that this peptide dose provided adequate systemic and liver bioavailability in mice and blocked phosphorylation of C/EBPβ-Thr217^21^,^27^. Animals were sacrificed at 30 hr at the peak of liver injury.

**Figure 3:**
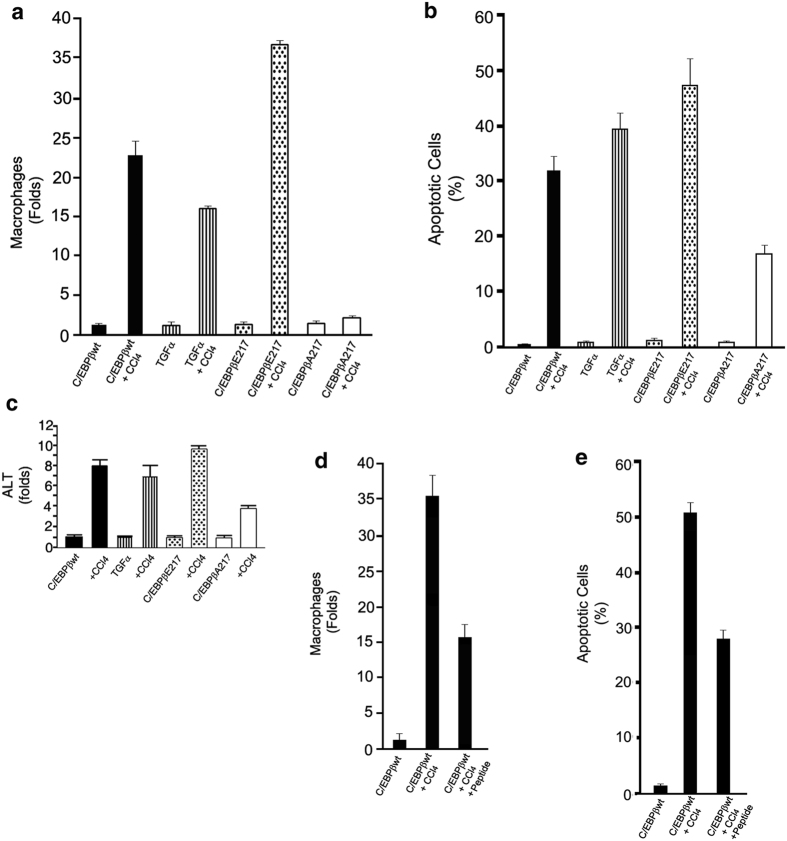
Phosphorylation of C/EBPβ on Thr217 is induced and necessary for the liver macrophage activation after hepatotoxin treatment in mice. (**a**) Acute administration of CCl_4_ stimulated a higher degree of macrophage infiltration in the livers of the phosphorylation mimic C/EBPβ-Glu217 mice compared to C/EBPβ-wt mice (*P* < 0.0001), as identified by the expression of F4/80 by microscopy. The C/EBPβ-Ala217 transgene suppressed CCl_4_-induced macrophage liver infiltration by about 90% when compared to C/EBPβ-wt mice (*P* < 0.0001). CCl_4_-induced macrophage liver infiltration was similar in TGFα transgenic mice and C/EBPβ-wt mice (NS). (**b)** The degree of hepatocyte apoptosis induced by CCl_4_ was increased in C/EBPβ-Glu217 mice (*P* < 0.005) and in TGFα mice (*P* < 0.05) but it was ameliorated in C/EBPβ-Ala217 mice (*P* < 0.01) when compared to C/EBPβ-wt mice. (**c)** CCl_4_ stimulated higher serum ALT in C/EBPβ-Glu217 mice compared to C/EBPβ-wt mice (*P* < 0.01). The C/EBPβ-Ala217 transgene suppressed CCl_4_-induced serum ALT by about 50% when compared to C/EBPβ-wt mice (*P* < 0.001). CCl_4_-induced serum ALT was similar in TGFα transgenic mice and C/EBPβ-wt mice (NS). (**d)** The dominant negative peptide that blocks C/EBPβ-Thr217 phosphorylation also inhibited the CCl_4_-induction of liver macrophage infiltration by ~60% (*P* < 0.01). (**e)** The peptide inhibited the CCl_4_-induction of hepatocyte apoptosis by ~45% (*P* < 0.001). Values are mean (SD) of at least 6 animals per group and representative of two experiments.

CCl_4_ treatment induced a severe acute liver injury with architectural collapse in C/EBPβ-wt mice but a mild-to-moderate injury in C/EBPβ-Ala217 mice (Supplementary Fig. 3a; reticulin stain). As we found for Fas (Fig. 1a and Supplementary Fig. 1), the liver injury induced by CCl_4_ was also more severe in C/EBPβ-Glu217 mice (Fig. 3 and Supplementary Fig. 3a). The degree of liver injury by histological analysis in these animal models correlated with both macrophage infiltration of the liver (Fig. 3a and Supplementary Fig. 3a; F4/80 stain), the degree of hepatocyte apoptosis (Fig. 3b), and the serum ALT levels (Fig. 3c).

Acute administration of CCl_4_ stimulated ~20-fold macrophage infiltration of the liver in C/EBPβ-wt mice after 30 hr (*P* < 0.005), as identified by the expression of F4/80 by quantitative microscopy^23^ (Fig. 3a). CCl_4_ administration induced even a higher degree of macrophage infiltration in the livers of the phosphorylation mimic C/EBPβ-Glu217 mice (~40-folds) (*P* < 0.0001) (Fig. 3a). Moreover, blocking phosphorylation of C/EBPβ-Thr217 with the C/EBPβ-Ala217 transgene suppressed CCl_4_-induced macrophage liver infiltration by about 90% when compared to C/EBPβ-wt mice (*P* < 0.001) (Fig. 3a).

The dominant negative peptide that blocks C/EBPβ-Thr217 phosphorylation^21^, also inhibited the CCl_4_-induction of liver macrophage infiltration by ~60% (*P* < 0.01) (Fig. 3d and Supplementary Fig. 3b; F4/80 stain) as well as liver injury by ~45% (*P* < 0.001) (Fig. 3e and Supplementary Fig. 3b; reticulin stain).

### Macrophages are induced and necessary for the liver injury in response to hepatotoxin treatment in mice

**Figure 4:**
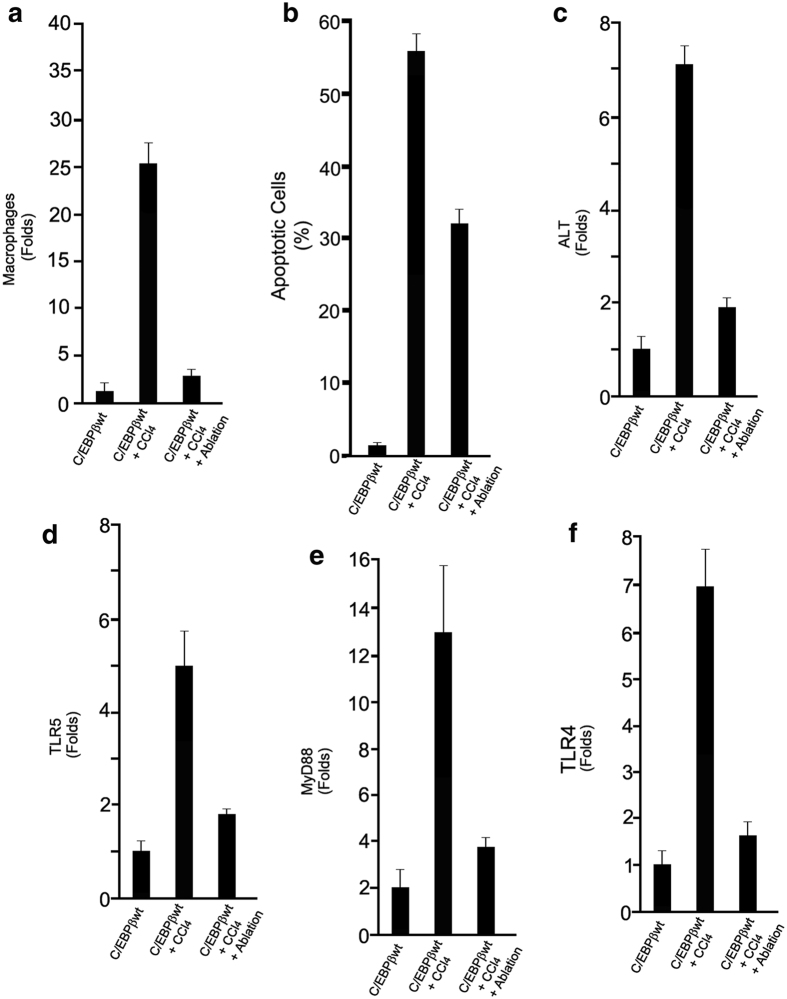
Macrophages are induced and necessary for the liver injury in response to hepatotoxin treatment in mice. (**a**) C/EBPβ-wt mice that received Clodronate liposomes to deplete macrophages 24 hr before the administration of CCl_4_, had a marked reduction in liver macrophages 30-hr after CCl_4_ treatment (~90%; *P* < 0.005). (**b)** Depletion of macrophages with Clodronate liposomes in C/EBPβ-wt mice resulted in decreased liver injury at 30-hr after CCl_4_ treatment as assessed by counting apoptotic hepatocytes in liver biopsies (*P* < 0.01). (**c)** Clodronate liposomes pretreatment of C/EBPβ-wt mice also decreased serum ALT levels by ~75% at 30-hr after CCl_4_ treatment (*P* < 0.005). (**d–f).** Clodronate liposomes induced an inhibition of TLR5, MyD88 and TLR4 expression in liver macrophages isolated from C/EBPβ-wt mice at 30-hr after CCl_4_ treatment compared to liver macrophages isolated from CCl_4_ treated C/EBPβ-wt mice that did not receive Clodronate liposomes (*P* < 0.001). Values are mean (SD) of at least 6 animals per group and representative of two experiments.

To ascertain the role of macrophages in toxic liver injury with an alternative approach, C/EBPβ-wt mice received Clodronate liposomes to deplete macrophages 24 hr before the administration of the hepatotoxin^5^. These animals had a marked reduction in liver macrophages infiltration (~90%; *P* < 0.005) (Fig. 4a and Supplementary Fig. 4), and in liver injury at 30-hr after CCl_4 _treatment as assessed by counting apoptotic hepatocytes in liver biopsies (*P* < 0.01) (Fig. 4b and Supplementary Fig. 4) and by the measurement of serum ALT (~75%; *P* < 0.005) (Fig. 4c).

Thirty-hours after CCl_4_ treatment, the CD-11/CD-68 mouse macrophages purified from livers of C/EBPβ-wt mice expressed high levels of TLR5, MyD88 and TLR4 (Fig. 4d–f), which are critical components of the inflammasome^1^. As expected, Clodronate liposomes induced an inhibition of TLR5, MyD88 and TLR4 expression in liver macrophages isolated from CCl_4_ treated animals compared to liver macrophages isolated from CCl_4_ treated animals that did not receive Clodronate liposomes (*P* < 0.001) (Fig. 4d–f), suggesting that activation of the inflammasome in liver macrophages is relevant for the liver injury induced by the hepatotoxin. Altogether, the results obtained from experiments with phosphorylation dominant positive and dominant negative C/EBPβ-Thr217 transgenic mice and hepatocytes as well as with macrophage ablation suggest that phosphorylation of C/EBPβ-Thr217 (or C/EBPβ-Glu217) in macrophages is a critical step in hepatotoxin-induced liver injury.

### Phosphorylated C/EBPβ-Thr217 stimulates the inflammasome signal 1 complex in liver macrophages in mice

A priming stimulus (signal 1), acting through NFκB pathway, often precedes assembly of the inflammasome complex in order to upregulate the expression of pro-IL-1β and NALP3. Upon either ligand sensing or enzymatic activation within the cytosol (signal 2), the cytosolic sensors oligomerize to form an activation platform for caspase 1^32^.

**Figure 5:**
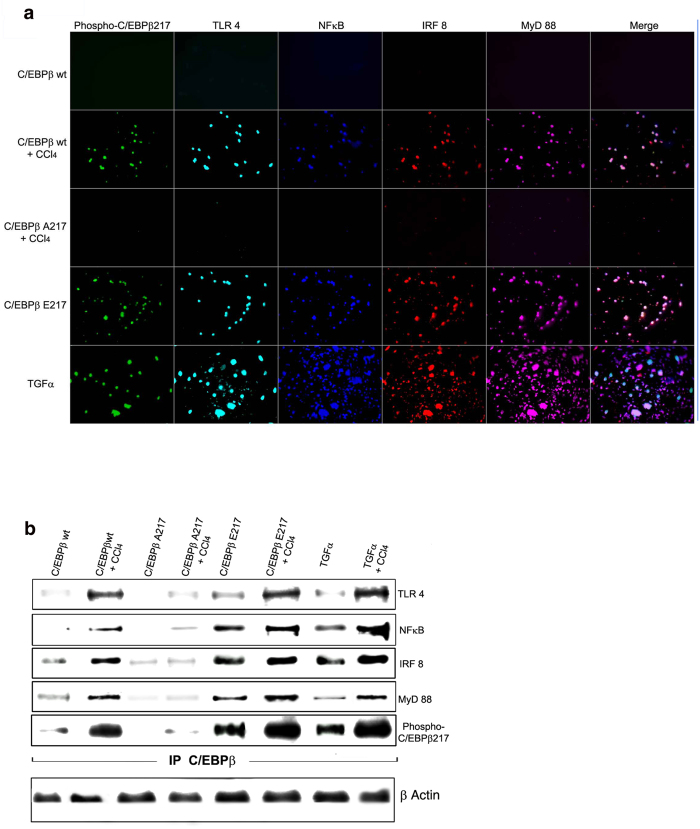
Phosphorylated C/EBPβ-Thr217 stimulates the inflammasome signal 1 complex in liver macrophages in mice. **(a)** Thirty-hours after CCl_4_ treatment, the CD-11/CD-68 primary liver macrophages isolated from C/EBPβ-wt mice expressed phosphorylated C/EBPβ-Thr217 and inflammasome signal 1 complex gene products, TLR4, NFκB, IRF8 and MyD88. Expression of phosphorylated C/EBPβ-Thr217, TLR4, NFκB, IRF8 and MyD88 was blocked in C/EBPβ-Ala217 transgenic mice. Liver macrophages isolated from C/EBPβ-Glu217 transgenic mice expressed the inflammasome signal 1 complex in the absence of CCl_4_ treatment, while liver macrophages isolated from TGFα mice expressed phosphorylated C/EBPβ-Thr217, TLR4, NFκB, IRF8 and MyD88 in the absence of CCl_4_ treatment. (*P* < 0.05 for C/EBPβ-wt mice treated with CCl_4_; C/EBPβ-Glu217 mice; and TGFα mice). Fluorescence and bright-field imaging were quantified using the Keyence microscope BZ9000 analysis software programs. Representative examples of three independent experiments described in legend to Fig. 3. (**b)** We immunoprecipitated C/EBPβ and analyzed its associated proteins from freshly isolated primary liver macrophages 30 hr after treatment of mice with vehicle or CCl_4_. Phosphorylated C/EBPβ-Thr217 (or C/EBPβ-Glu217), but not unphosphorylated C/EBPβ-Thr217 (or C/EBPβ-Ala217), was associated with TLR4, NFκB, IRF8 and MyD88. Treatment with CCl_4_ (and macrophage activation) increased the association between phosphorylated C/EBPβ-Thr217 and inflammasome signal 1 proteins. β-Actin was use as internal control for sample loading. Representative examples of three independent experiments described in legend to Fig. 3.

Thirty-hours after CCl_4_ treatment, the CD-11/CD-68 primary liver macrophages purified from C/EBPβ-wt mice expressed phosphorylated C/EBPβ-Thr217, which was co-expressed with critical components of the inflammasome signal 1 complex gene products, including TLR4, NFκB, IRF8 and MyD88 (Fig. 5a)^1^.

As expected, phosphorylation of C/EBPβ-Thr217 is required for the expression of the inflammasome signal 1 complex in liver macrophages induced by hepatotoxin treatment since it was blocked in the nonphosphorylatable C/EBPβ-Ala217 transgenic mice (Fig. 5a). In contrast, liver macrophages isolated from the dominant positive C/EBPβ-Glu217 transgenic mice expressed the inflammasome signal 1 complex even in the absence of hepatotoxin treatment (Fig. 5a). Similarly, in liver macrophages isolated from TGFα transgenic mice, which have an stimulated MAPK signaling, phosphorylated C/EBPβ-Thr217 was associated with the expression of critical protein components of the inflammasome signal 1 complex, including TLR4, NFκB, IRF8 and MyD88 (Fig. 5a).

To further delineate the physical association of phosphorylated C/EBPβ-Thr217 with members of the inflammasome signal 1 complex in purified liver macrophages after hepatotoxin treatment, we immunoprecipitated C/EBPβ, which was normalized by β-actin for the immunoblots, and analyzed its associated proteins. We found that phosphorylated C/EBPβ-Thr217 or C/EBPβ-Glu217, but not unphosphorylated C/EBPβ-Thr217 or C/EBPβ-Ala217, were physically associated with TLR4, NFκB, IRF8 and MyD88 in freshly isolated primary liver macrophages (Fig. 5b). Treatment with CCl_4_ (and the consequent macrophage activation) increased the association between phosphorylated C/EBPβ-Thr217 or C/EBPβ-Glu217 and inflammasome signal 1 proteins (Fig. 5b).

### Phosphorylated C/EBPβ-Thr217 stimulates expression of the inflammasome complex signal 2 in liver macrophages in mice

**Figure 6:**
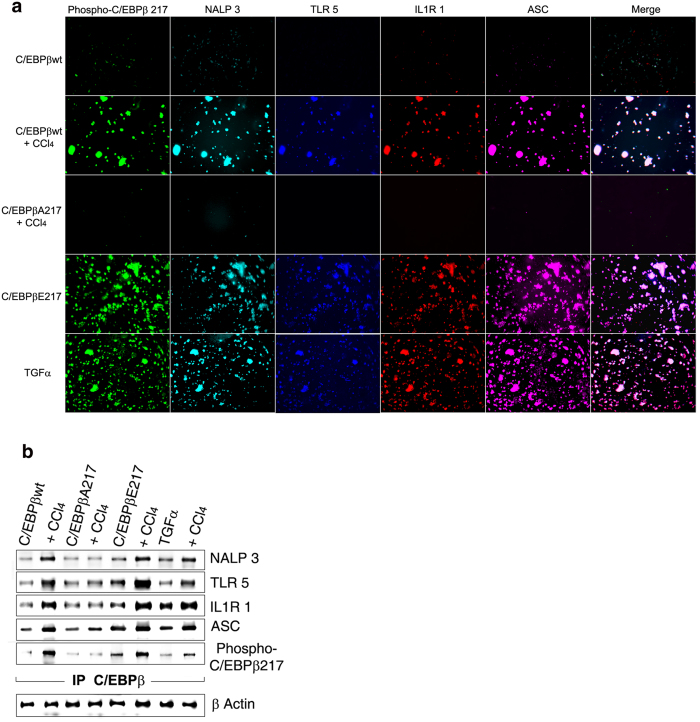
Phosphorylated C/EBPβ-Thr217 stimulates expression of the inflammasome complex signal 2 in liver macrophages in mice. (**a**) Thirty-hours after CCl_4_ treatment, the CD-11/CD-68 primary liver macrophages purified from C/EBPβ-wt mice expressed phosphorylated C/EBPβ-Thr217 and inflammasome signal 2 complex gene products, NALP3, TLR5, IL-1R1 and the adaptor protein ASC. Expression of phosphorylated C/EBPβ-Thr217, NALP3, TLR5, IL-1R1 and the adaptor protein ASC was blocked in C/EBPβ-Ala217 transgenic mice. Liver macrophages isolated from C/EBPβ-Glu217 transgenic mice expressed the inflammasome signal 2 complex in the absence of CCl_4_ treatment, while liver macrophages isolated from TGFα mice expressed phosphorylated C/EBPβ-Thr217, NALP3, TLR5, IL-1R1 and the adaptor protein ASC in the absence of CCl_4_ treatment. (*P* < 0.01 for C/EBPβ-wt mice treated with CCl_4_; C/EBPβ-Glu217 mice; and TGFα mice). Fluorescence and bright-field imaging were quantified using the Keyence microscope BZ9000 analysis software programs. Representative examples of three independent experiments described in legend to Fig. 3. (**b)** We immunoprecipitated C/EBPβ and analyzed its associated proteins from freshly isolated primary liver macrophages 30 hr after treatment of mice with vehicle or CCl_4_. Phosphorylated C/EBPβ-Thr217 (or C/EBPβ-Glu217), but not unphosphorylated C/EBPβ-Thr217 (or C/EBPβ-Ala217), was associated with NALP3, TLR5, IL-1R1 and the adaptor protein ASC. Treatment with CCl_4_ (and macrophage activation) increased the association between phosphorylated C/EBPβ-Thr217 and inflammasome signal 2 proteins. β-Actin was use as internal control for sample loading. Representative examples of three independent experiments described in legend to Fig. 3.

Given that activation of the inflammasome signal 2 pathway is essential for expression of several inflammatory cytokines^1^,^11^,^33^, we analyzed the role of phosphorylated C/EBPβ-Thr217 on the inflammasome signal 2 pathway in liver macrophages. We found that CCl_4_ treatment of C/EBPβ-wt mice stimulated the expression of the inflammasome signal 2 proteins in liver macrophages (Fig. 6a). Thirty-hours after CCl_4_ treatment, the CD-11/CD-68 liver macrophages freshly purified from C/EBPβ-wt mice expressed phosphorylated C/EBPβ-Thr217, which was co-expressed with critical components of the inflammasome complex signal 2, including NALP3, TLR5, IL-1R1 and the adaptor protein ASC (Fig. 6a)^1^. Expression of phosphorylated C/EBPβ-Thr217 is also required for the induction of the inflammasome multiprotein complex signal 2 in liver macrophages stimulated by hepatotoxin treatment since both were blocked in the nonphosphorylatable C/EBPβ-Ala217 mice (Fig. 6b). In contrast, liver macrophages isolated from the phosphorylation mimic C/EBPβ-Glu217 mice, even in the absence of hepatotoxin treatment, expressed a partially activated (primed) inflammasome signal 2 complex (Fig. 6a). In addition, in liver macrophages isolated from TGFα transgenic mice, phosphorylated C/EBPβ-Thr217 was associated with the expression of critical components of the inflammasome signal 2 complex, including NALP3, TLR5, IL-1R1 and ASC (Fig. 6a).

To further delineate the physical association of phosphorylated C/EBPβ-Thr217 with members of the inflammasome signal 2 complex in purified primary liver macrophages after hepatotoxin treatment, we immunoprecipitated C/EBPβ and analyzed its associated proteins. We found that liver injury increased the physical association between inflammasome signal 2 complex proteins (NALP3, TLR-5, IL-1R1 and ASC) in liver macrophages with phosphorylated C/EBPβ-Thr217 or C/EBPβ-Glu217, but not with unphosphorylated C/EBPβ-Thr217 or C/EBPβ-Ala217 (Fig. 6b).

### Phosphorylated C/EBPβ-Thr217 stimulates the expression of inflammasome structural and byproduct genes in liver macrophages in mice

**Figure 7:**
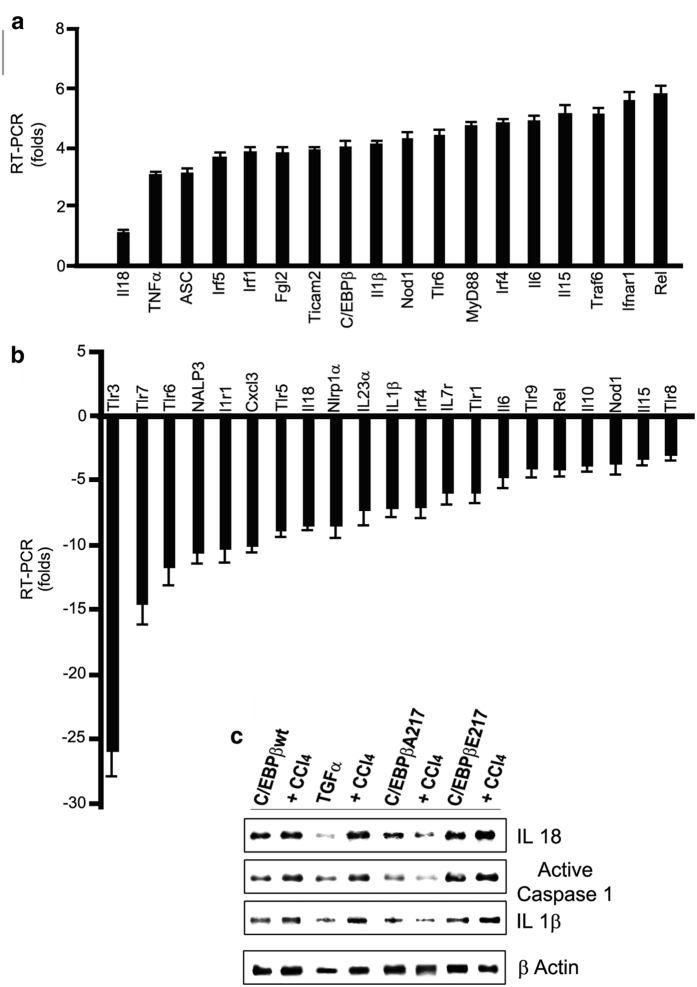
Phosphorylated C/EBPβ-Thr217 stimulates the expression of inflammasome structural and byproduct genes in liver macrophages in mice. (**a)** Freshly isolated liver macrophages from the phosphorylation mimic C/EBPβ-Glu217 mice expressed an activated transcriptosome related to the Inflammasome when compared to C/EBPβ-wt mice. This included the increased expression of inflammasome genes (ASC, IRF-1, IRF-4, IRF-5, TCAM-2, TLR-6, TRAF-6, MyD-88, Nod-1 and Rel) as well as the increased expression of direct and indirect cytokine byproducts (IL-1β, IL-6, IL-15, IL-18 and TNFα). (**b**) Freshly isolated C/EBPβ-Ala217 liver macrophages from mice treated with CCl_4_ express an inhibited inflammasome transcriptosome when compared to freshly isolated liver macrophages from C/EBPβ-wt mice treated with CCl_4_. This included the decreased expression of inflammasome genes (IRF-4, NALP-α, NALP-3, TCAM-2, TLR-1, TLR-3, TLR-5, TLR-6, TLR-7, TLR-8, TLR-9, Nod-1 and Rel) as well as the decreased gene expression of direct and indirect cytokine inflammasome byproducts (IL-1β, IL-6, IL-10, IL-15, IL-18, IL-23α and CXCL-3). (**c**) Treatment with CCl_4_ was associated with the induction of IL-18, active caspase-1 and IL-1β inflammasome protein expression in the livers of C/EBPβ-wt, C/EBPβ-Glu217, and TGFα mice. Values are mean (SD) of triplicates and representative of three experiments.

We found that freshly isolated liver macrophages from the phosphorylation mimic C/EBPβ-Glu217 mice express an activated transcriptosome related to the Inflammasome when compared to C/EBPβ-wt mice. This included the increased expression of inflammasome genes (ASC, IRF-1, IRF-4 IRF-5, TCAM-2, TRL-6, TRAF-6, Myo-D88, Nod-1 and Rel) as well as the increased expression of direct and indirect cytokine gene byproducts (IL-1β, IL-6, IL-15, IL-18 and TNFα)^1^,^11^,^33^ (Fig. 7a). These data suggest that phosphorylated C/EBPβ-Thr217 (or C/EBPβ-Glu217) is required for the expression of the inflammasome structural proteins and byproducts. Further, freshly isolated C/EBPβ-Ala217 liver macrophages from mice treated with CCl_4_ express an inhibited inflammasome transcriptosome when compared to freshly isolated liver macrophages from C/EBPβ-wt mice treated with CCl_4_. This included the decreased expression of inflammasome genes (IRF-4, NALP-α, NALP-3, TCAM-2, TRL-1, TRL-3, TRL-5, TRL-6, TRL-7, TRL-8, TRL-9, Nod-1 and Rel) as well as the decreased expression of direct and indirect cytokine inflammasome gene byproducts (IL-1β, IL-6, IL-10, IL-15, IL-18, IL-23α and CXCL-3)^1^,^11^,^33^ (Fig. 7b). In addition, treatment with CCl_4_ was associated with the induction of IL-18, active caspase-1 and IL-1β inflammasome protein expression^1^ in the livers of C/EBPβ-wt, C/EBPβ-Glu217, and TGFα mice (Fig. 7c).

### Human liver injury induced by the Toxic Oil Syndrome is also characterized by phosphorylated C/EBPβ-Thr266 associated with the inflammasome complex in liver macrophages

**Figure 8:**
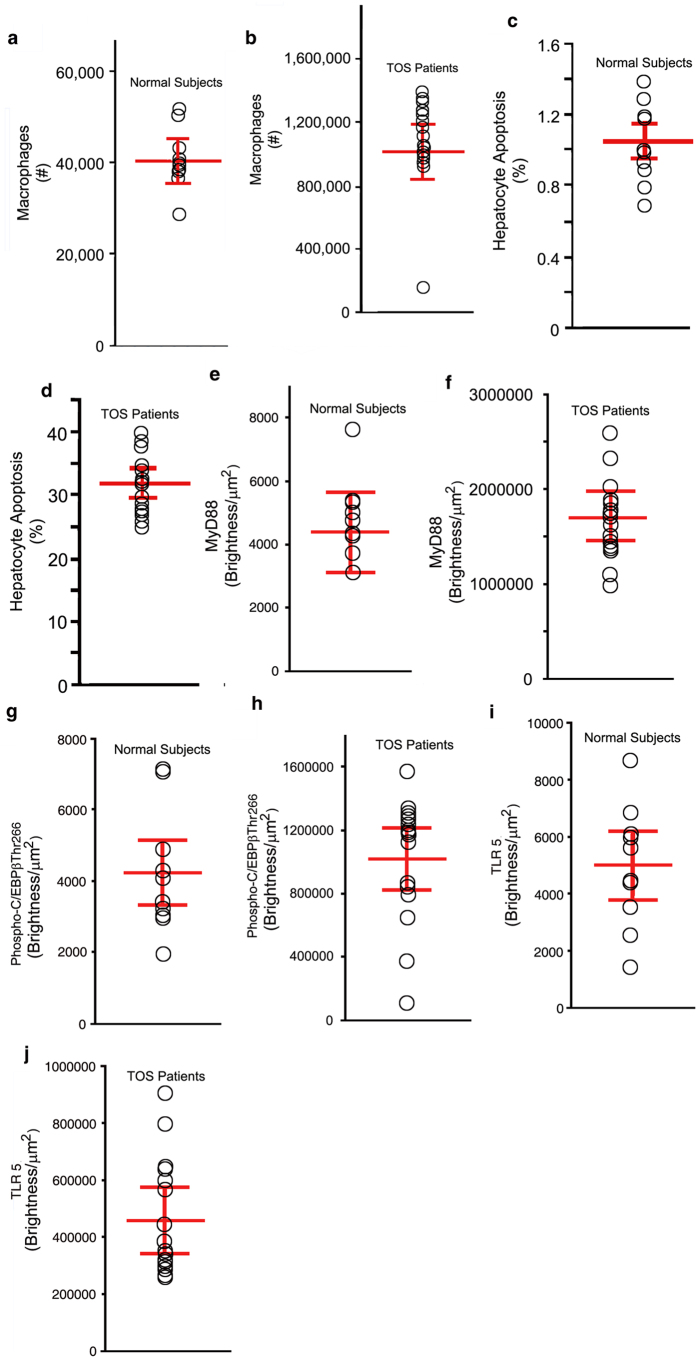
C/EBPβ-Thr266 associated with the inflammasome complex in liver macrophages from patients with Toxic Oil Syndrome. We analyzed liver biopsies from all 16 patients with TOS that were still available at the Universidad Complutense Medical Center, Madrid, Spain. These patients had a moderately severe acute liver injury. (**a**,**c)** In TOS patients there was a marked increase in both macrophage infiltration of the liver (~20-fold; 1,004,683 +/− 140,485 vs. 41,160 +/− 3,353; *P* < 0.001) (**a**) and the degree of hepatocyte apoptosis (~30-fold; 32.0 +/− 4.7% vs. 1.0 +/− 0.2%; *P* < 0.001) (**c**) compared to normal subjects (**b**,**d**). (**f**,**h**,**j**). Liver macrophages in livers from patients with TOS expressed MyD-88, phosphorylated C/EBPβ-Thr266, and TLR-5 when compared to macrophages in normal livers (**e**,**g**,**i**) (*P* < 0.001 for all).

The Toxic Oil Syndrome (TOS) that occurred in central and northwestern Spain in the summer of 1981 affected approximately 20,000 people, whom were afflicted with acute liver injury. The oxidative stress liver injury was induced in a dose-response manner by the olive oil contaminant 1, 2-dioleoyl ester of 3-(*N*-phenyl amino)-1, 2-propanediol^34^,^35^. We analyzed liver biopsies from all 16 patients with TOS that were still available at the Universidad Complutense Medical Center, Madrid, Spain. These patients had a moderately severe acute liver injury as characterized by the elevated ALT and aspartate aminotransferase (AST) with a cholestastic component judging by the increased alkaline phosphatase and total bilirubin, when compared to normal individuals (Supplementary Table 1). The degree of liver injury by histological analysis in these TOS patients correlated with both macrophage infiltration of the liver (Fig. 8b), the degree of hepatocyte apoptosis (Fig. 8d), compared to control (Fig. 8a,c) and the serum ALT levels (Supplementary Table 1).

Because TOS is characterized by oxidative stress that results in an acute inflammatory liver injury, we analyzed whether the livers of TOS afflicted patients had similar features to the CCl_4_ animal models with acute inflammatory liver injury. Liver macrophages, characterized by the expression of specific markers as described above, in livers from patients with TOS expressed phosphorylated C/EBPβ-Thr266 (the exact homologue of mouse Thr217) when compared to macrophages in normal livers (Fig. 8g,h). We found that the TOS livers have increased markers characteristic of the activated inflammasome, MyD-88 and TLR-5 (Fig. 8f,j) when compared to macrophages in normal livers (Fig. 8e,i).

## Discussion

In this study we have found a novel role of phosphorylated C/EBPβ-Thr217 in the activation of the inflammasome in liver macrophages, resulting in amplification of the liver injury induced by CCl_4_ or by Fas-L. C/EBPβ-Thr217 phosphorylation is required for macrophage infiltration of the liver after a liver injury induced in mice by the oxidative stress hepatotoxin CCl_4_ or by Fas-L, and for macrophage activation in primary liver macrophage cultures (stimulated by TGFα, an inducer of C/EBPβ-Thr217 phosphorylation^18^).

Remarkably, blocking the phosphorylation of C/EBPβ-Thr217 by expressing a dominant negative non-phosphorylatable C/EBPβ-Ala217 transgene in mice or by administering an inhibitory peptide of C/EBPβ phosphorylation to C/EBPβ-wt mice prevented the liver injury induced by CCl_4_ or by Fas-L. Inhibiting the phosphorylation of C/EBPβ-Thr217 also ameliorated macrophage liver infiltration, expression and activation of the inflammasome multiprotein complex as well as the polarization of pro-inflammatory liver macrophages. We have previously shown that spleen macrophages *in vivo* in C/EBPβ-Ala217 transgenic mice had increased caspase 3 expression (suggestive of activated apoptosis pathways) compared to control mice^28^. However, in this study, we do not find any decrease in liver macrophage numbers in C/EBPβ-Ala217 transgenic mice compared to control mice (Fig. 3a). This strongly suggests that in the liver, unlike in the spleen, there is no increased apoptosis of C/EBPβ-Ala217 macrophages. The apparent differences in macrophage apoptosis and survival in spleen and liver is of interest and it will require a more extensive analysis to elucidate the mechanisms.

Specifically, phosphorylation of C/EBPβ-Thr217 in liver macrophages was required for stimulating the expression of the multiprotein complex inflammasome signal 1 (NFκB, IRF8, the adaptor protein MyD88 and TLR4) and of the inflammasome signal 2 pathway (NALP3, TLR5, IL-1R1 and the adaptor protein ASC)^1^,^11^,^33^. Phosphorylated C/EBPβ-Thr217, but not unphosphorylated C/EBPβ-Thr217, was also found to be physically associated with the inflammasome multiprotein complex signal 1 and signal 2.

The central component of an inflammasome is a member of the NALP family, and this protein associates with the adaptor protein apoptosis-associated speck-like protein (ASC), which in turn recruits pro-inflammatory-caspase precursors (such as pro-caspase-1)^36^. NALP3, which we found in our model of inflammasome activation in liver macrophages, is able to form inflammasomes while mutations in the gene that encodes NALP3 (*CIAS1*) cause several auto-inflammatory disorders, indicating its physiological relevance^36^.

An acute oxidative stress liver injury with CCl_4_ in the phosphorylated mimic C/EBPβ-Glu217 mice induced the expression of liver macrophage inflammasome genes (ASC, IRF-1, IRF-4 IRF-5, TCAM-2, TRL-6, TRAF-6, Myo-D88, Nod-1 and Rel) as well as the increased gene expression of direct and indirect cytokine inflammasome byproducts of liver macrophages (IL-1β, IL-6, IL-10, IL-15, IL-18, IL-23α and CXCL-3), a hallmark of inflammasome activation^1^,^11^,^33^,^37^. In contrast, freshly isolated nonphosphorylatable C/EBPβ-Ala217 liver macrophages from mice treated with CCl_4_ expressed an inhibited inflammasome transcriptosome when compared to freshly isolated liver macrophages from C/EBPβ-wt mice treated with CCl_4_. This included the decreased expression of inflammasome genes (IRF-4, NALP-α, NALP-3, TCAM-2, TRL-1, TRL-3, TRL-5, TRL-6, TRL-7, TRL-8, TRL-9, Nod-1 and Rel) as well as the decreased gene expression of direct and indirect cytokine inflammasome byproducts (IL-1β, IL-6, IL-10, IL-15, IL-18, IL-23α and CXCL-3).

The C/EBPβ-Ala217 mutant functions as a *trans*-dominant negative of the C/EBPβ-Thr217 phosphorylation^21^. In contrast, the C/EBPβ-Glu217 mutant functions as a *trans*-dominant positive of the C/EBPβ-Thr217 phosphorylation^18^. It remains to be determined whether in liver injury, phosphorylation of C/EBPβ-Thr217 in macrophages stimulates macrophage proliferation/survival as reported for the Anthrax lethal toxin^23^ and/or facilitates migration to and direct destruction or phagocytosis of the injured hepatocytes. CCl_4_ increased the liver macrophage infiltration by ~20-fold, while the macrophage stimulating factor TGFα in transgenic mice, which lack hepatocyte injury, did not increase macrophage liver infiltration. We observed also macrophage infiltration in the livers of animals treated acutely with Fas-L, suggesting that regardless of the mechanisms of liver injury, the stimulation of C/EBPβ-Thr217 phosphorylation in macrophages modulates the infiltration of the liver by these cells.

The Fas-L experiments are physiologically relevant since significant elevations of soluble Fas-L occur in patients with drug-induced liver injury or alcoholic liver disease^38^,^39^. In excellent agreement with the main hypothesis, acute FasL administration (acting on TNF superfamily receptors) induced greater macrophage infiltration, and liver injury in the phosphorylation mimic C/EBPβ-Glu217 transgenic mice. Mice expressing the C/EBPβ-Ala217 transgene were refractory to development of liver injury by Fas-L.

Modulation of macrophage activity by ablation also indicated the essential role of phosphorylated C/EBPβ-Thr217 in macrophages for the induction of liver injury after hepatotoxin exposure. We have reported that amplification of toxic liver injury is mediated by macrophages since TLR-4 ko mice were resistant to hepatotoxins and that reconstitution of bone marrow irradiated TLR-4 ko mice with TLR-4^+/+^ macrophages conferred susceptibility of these animals to hepatotoxins^4^. More recently, the role of macrophages has been confirmed in toxic liver injury using macrophage ablation^5^, in an experimental alcoholic liver injury model using an IL-1 receptor antagonist^6^, and in LPS/D-galactosamine induced liver injury using Adenosine-_2A_ (A_2A_) receptor-ko mice^7^. Adenosine is required for sustained inflammasome activation via the A2A receptor and the HIF-1α pathway^7^. In addition, both A_2A_ adenosine receptors and C/EBPβ are required for IL-10 production by macrophages exposed to *Escherichia coli*^40^, suggesting a potential convergence of phosphorylated C/EBPβ-Thr217, as characterized first in the present study, and the A_2A_ signaling pathways in activated liver macrophages.

Our current studies characterized phosphorylated C/EBPβ-Thr217 in macrophages as a novel and major signaling pathway in hepatotoxin-induced liver injury. It remains to be determined whether phosphorylated C/EBPβ-Thr217 (human Thr266) also plays a major role in the macrophage inflammasome in liver injury induced by experimental and human alcoholic and non-alcoholic steatohepatitis (NASH)^1^,^41^.

Our findings are consistent with the role of C/EBPβ as a critical signaling protein for macrophages since expression of a dominant inhibitor of C/EBPβ DNA-binding sites^15^ or of a targeted deletion of C/EBPβ results in impaired macrophage differentiation^16^.

The features of a well characterized acute human oxidative stress liver injury, the Toxic Oil Syndrome (TOS), which was induced by a toxic contaminant, mimics and validates, at least in part, our findings with animal models of acute oxidative stress inflammatory liver injury. Our findings in human acute liver injury due to TOS suggest that our findings in cellular and animal models may be applicable to some types of acute liver injury in humans. Future studies to understand these pathways in human acute liver injury may define whether or not phosphorylated C/EBPβ-Thr266 in macrophages is pathogenic in those injuries.

In brief, our findings provide a novel signaling mechanism through C/EBPβ-Thr217 (human Thr266) for the inflammasome multiprotein complex activation in liver macrophages as a critical step for the development of liver inflammation and injury^1^.

Liver inflammation and injury are major contributors to the morbidity and mortality of acute and chronic liver diseases in humans^1^,^2^,^3^,^41^. Thus, IL-1β receptor antagonists^6^, A_2A_ receptor antagonists^7^, and small molecule peptido mimetics, as targeted inhibitors of human C/EBPβ-Thr266 phosphorylation, in liver macrophages may be potential candidates for the prevention and treatment of inflammatory liver injury.

## Methods

The methods were carried out in accordance with the approved guidelines.

### Construction of C/EBPβ-Ala217 and C/EBPβ-Glu217 mice

The Animal Protocol was approved by the VA San Diego Healthcare System’s Veterinarian Medical Unit. Transgenic mice expressing either the C/EBPβ-Ala217, a dominant negative, nonphosphorylatable mutation, or C/EBPβ-Glu217, a dominant positive, phosphorylation mimic mutation of the C/EBPβ-Thr217 phosphoacceptor, were generated as described previously^27^ and back-crossed to the parental wild-type inbreed FVB mice for >10 generations. The presence of the *rsv* gene was used to identify these transgenic mice by PCR. The primer sequences for the RSV PCR were custom designed (RSV.2271 TAGGGTGTGTTTAGGCGAAA sense and RSV.2510 TCTGTTGCCTTCCTAATAAG antisense).

### Animal Procedures

In the acute exposure to the hepatotoxins, C/EBPβ-wt, C/EBPβ-Ala217 and C/EBPβ-Glu217 mice^27^ (23–27 g) each received intraperitoneal injections of CCl_4_ (70 μl CCl_4 _and 30 μl of mineral oil) or mineral oil (70 μl saline and 30 μl mineral oil), or Jo-2 Ab (Fas-L; 0.2 μg/g body weight) or saline vehicle (50 μl) only once. In other experiments, C/EBPβ-wt mice (25 g) each received intraperitoneal injections of CCl_4_ (70 μl CCl_4_ and 30 μl of mineral oil) or mineral oil (70 μl saline and 30 μl mineral oil) once but after 8 hr animals received either 50 μl saline (vehicle) or the cell permeant Ac-KAla217VD-CHO^21^ (American Peptide) (100 μg IP). In these experiments, animals were sacrificed 30 hr after the last CCl_4_ injection or 8 hr after the Fas-L injection.

### Macrophage purification

We followed the reported standards for *in vitro* experiments with macrophages^42^. Adult C/EBPβ -wt (yield 1.3 × 10^5 ^macrophages per liver), C/EBPβ-Ala217 (yield 2.2 × 10^5^ macrophages per liver), C/EBPβ-Glu217 (yield 1.4 × 10^5^ macrophages per liver) and TGFα (yield 1.6 × 10^5^ macrophages per liver) mice of FVB background were used for the isolation of primary liver macrophages. Cells were prepared, by *in situ* perfusion and single-step density Nycodenz gradient (Accurate Chemical & Scientific, Westbury, NY), as described previously^43^. Liver macrophages were isolated at density gradient of 13% and then affinity purified by magnetic beads linked to CD-11/CD-68 Antibodies (Miltenyl Biotechnology). No CSF-1 or supplements were used. An aliquot was plated on glass coverslips and allowed to sit 1 hr at 37 C and then fixed with acetone: methanol. Liver macrophages were identified by their typical morphology, adherence to glass, and with antibodies against F-4/80 and CD-68. Purity of these preparations was greater than 95%. Aliquot of macrophages were cultured in RPMI 1640, 10% fetal bovine serum with L-glutamine, 25 μM HEPES and Penicillin/Streptomycin. In some experiments, liver macrophages were treated for 8 hr with TGFα (10 μM).

### Microscopy

Fluorescent labels were observed using antibodies against C/EBPβ, RSKPhosphoSer380, F4/80, NOS-2 TLR4, NFκB, IRF8, MyD88, NALP3, TLR-5, IL-1R1 and ASC (Santa Cruz Biotechnology, Santa Cruz, California), or C/EBPβ-PhosphoThr217 in a Keyence fluorescent microscope fluorochromes utilized were Alexa 488, 750, 350, 647, and 594. At least 100 cells were analyzed per experimental point^18^,^21^,^23^,^27^. We used TO-PRO-3 (Molecular Probes, Eugene, Oregon) to analyze nuclear morphology. Fluorescence and bright-field imaging were quantified using the Keyence microscope BZ9000 analysis software programs. The inter-observer agreement was >90%.

### Inflammation Genes

The liver macrophage expression of 86 inflammation genes was determined by using the RT^2^ Quantitative Real-Time PCR Array as described by the manufacturer (SABiosciences; Valencia, CA). Control and experimental freshly isolated liver macrophage samples were analyzed together with internal control samples for the RNA purification and amplification steps, as well as for housekeeping genes (β-actin), using the Bio-Rad iQ5 real-time PCR detection system (Bio-Rad, Hercules, CA)^21^. Isolation of total RNA, treatment with DNase, precipitation with chloroform, and cDNA synthesis was performed using 1 μg of total RNA as described for RT-PCR following the manufacturer’s recommendations.

### Immunoprecipitation and Immunoblots

Pre-cleared freshly isolated liver macrophage cell lysates were incubated for 2 h with purified C/EBPβ antibodies followed by the addition of A/G+ agarose (Santa Cruz Biotechnology) for 12 h. The immunoprecipitation reactions each contained 500 μg of total protein and 2 μg antibody (or purified IgG pre-immune serum as negative control). Immunoprecipitates were washed 3 times in 500 ml cell lysis buffer and resolved by SDS-PAGE, and C/EBPβ-PhosphoThr217, TLR4, NFκB, IRF8, MyD88, NALP3, TLR-5, IL-1R1, ASC β-actin, active caspase 3, IL-1β and IL-18 detected by western blot, following the chemoluminescence protocol (Perkin-Elmer, Shelton, Connecticut) using specific antibodies^18^,^19^,^21^,^27^. Negative samples were performed omitting the first antibody.

### Human Livers

We obtained anonymous, de-identified liver samples from 16 patients with acute liver injury secondary to Toxic Oil Syndrome and moderately severe liver injury^35^ and from 10 control subjects without liver disease (NDRI). The protocol was approved by the University of San Diego, San Diego Human Protection Program.

### Statistical Analysis

Results are expressed as mean (±SD or ±SE). Either the Student-*t* or the Wilcoxon Mann-Whitney tests were used to evaluate the differences of the means between groups for parametric and non-parametric populations, respectively, with a *P* value of <0.05 as significant.

## Additional Information

**How to cite this article**: Buck, M. *et al.* C/EBPβ-Thr217 Phosphorylation Stimulates Macrophage Inflammasome Activation and Liver Injury. *Sci. Rep.* **6**, 24268; doi: 10.1038/srep24268 (2016).

## Supplementary Material

Supplementary Information
