# Supplementary figures and images for: C/EBPβ-Thr217 Phosphorylation Stimulates Macrophage Inflammasome Activation and Liver Injury
Source: Sci Rep. 2016 Apr 12;6:24268. doi: 10.1038/srep24268 (PMC4828658; doi:10.1038/srep24268)

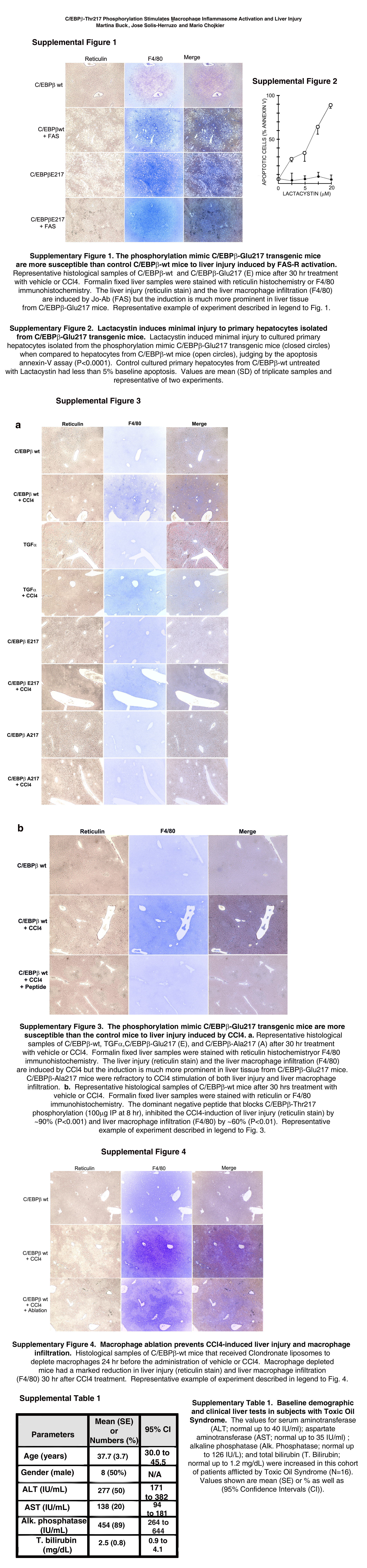

Supplement: Supplementary Information [file srep24268-s1.tiff]
